# The Impact of Leaving Group Anomericity on the Structure of Glycosyl Cations of Protected Galactosides

**DOI:** 10.1002/cphc.202000473

**Published:** 2020-07-30

**Authors:** Kim Greis, Eike Mucha, Maike Lettow, Daniel A. Thomas, Carla Kirschbaum, Sooyeon Moon, Alonso Pardo‐Vargas, Gert von Helden, Gerard Meijer, Kerry Gilmore, Peter H. Seeberger, Kevin Pagel

**Affiliations:** ^1^ Institute of Chemistry and Biochemistry Freie Universität Berlin Arnimallee 22 14195 Berlin Germany; ^2^ Department of Molecular Physics Fritz Haber Institute of the Max Planck Society Faradayweg 4–6 14195 Berlin Germany; ^3^ Max Planck Institute of Colloids and Interfaces Am Mühlenberg 1 14476 Potsdam Germany

**Keywords:** anomeric memory, IR spectroscopy, mass spectrometry, glycosylation, glycosyl cation

## Abstract

It has been reported that fragments produced by glycosidic bond breakage in mass spectrometry‐based experiments can retain a memory of their anomeric configuration, which has major implications for glycan sequencing. Herein, we use cryogenic vibrational spectroscopy and ion mobility‐mass spectrometry to study the structure of B‐type fragments of protected galactosides. Cationic fragments were generated from glycosyl donors carrying trichloroacetimidate or thioethyl leaving groups of different anomeric configuration. The obtained infrared signatures indicate that the investigated fragments exhibit an identical structure, which suggests that there is no anomeric memory in B‐type ions of fully protected monosaccharides.

## Graphical abstract

**Do you remember**? Some of the glycan fragments produced in mass spectrometers can retain a memory of their anomeric configuration. Herein, we use gas‐phase infrared spectroscopy to study the structure of B‐type fragments of protected galactosides. The infrared signatures indicate that the investigated fragments exhibit an identical structure.

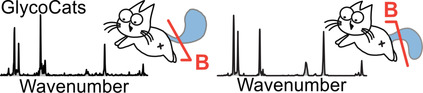

Glycans are responsible for crucial processes in living organisms.^1^ As a result, there is an increasing interest in using oligosaccharides as novel pharmaceuticals.^2^ In contrast to oligonucleotides and proteins, however, glycans are structurally highly diverse, with a complex branching, regio‐ and stereochemistry. This complexity represents the major challenge for glycan analysis and there is currently no gold standard method to comprehensively characterise complex oligosaccharides from the minute sample amounts that are commonly found in biology.

Gas‐phase infrared (IR) action spectroscopy and ion mobility‐mass spectrometry (IM‐MS) are useful to probe glycan structures and unravel mechanisms of chemical glycosylation reactions.^3^ Isomeric carbohydrates exhibiting minute structural variations can be distinguished based on their IR fingerprint or their IM‐MS derived collision cross section (CCS).^4^ Likewise, it recently became possible for the first time to comprehensively characterize the key intermediate in glycan synthesis, the short‐lived glycosyl cation, using infrared multiple photon dissociation (IRMPD) and cryogenic vibrational IR spectroscopy.^5^ Direct evidence for neighbouring and remote protecting group participation in glycosylation reactions was obtained. However, the impact of the leaving group and its anomericity (configuration) on the structure of glycosyl cations has not been fully elucidated yet.

Albeit discussed in a different context, it has furthermore been reported recently that certain fragment ions produced in MS experiments can retain stereochemical information of the glycosidic bond from which they originate. This effect, called anomeric memory, is based on the observation that the IRMPD spectra and/or the IM‐MS arrival time distributions of glycosidic B‐ and C‐fragments (Scheme 1) differ depending on the anomericity of the underlying glycosidic bond. For C‐fragments, the origin of the effect can be explained by the configuration of the anomeric carbon that is retained after fragmentation.^6^ In B‐type ions on the other hand, this observation is rather surprising at first glance, as the stereocentre at the anomeric carbon vanishes upon removal of a substituent. However, depending on the anomericity of the precursor, the fragments might adopt a distinct structure, which in turn would explain the reported differences.^7^

**Scheme 1:**
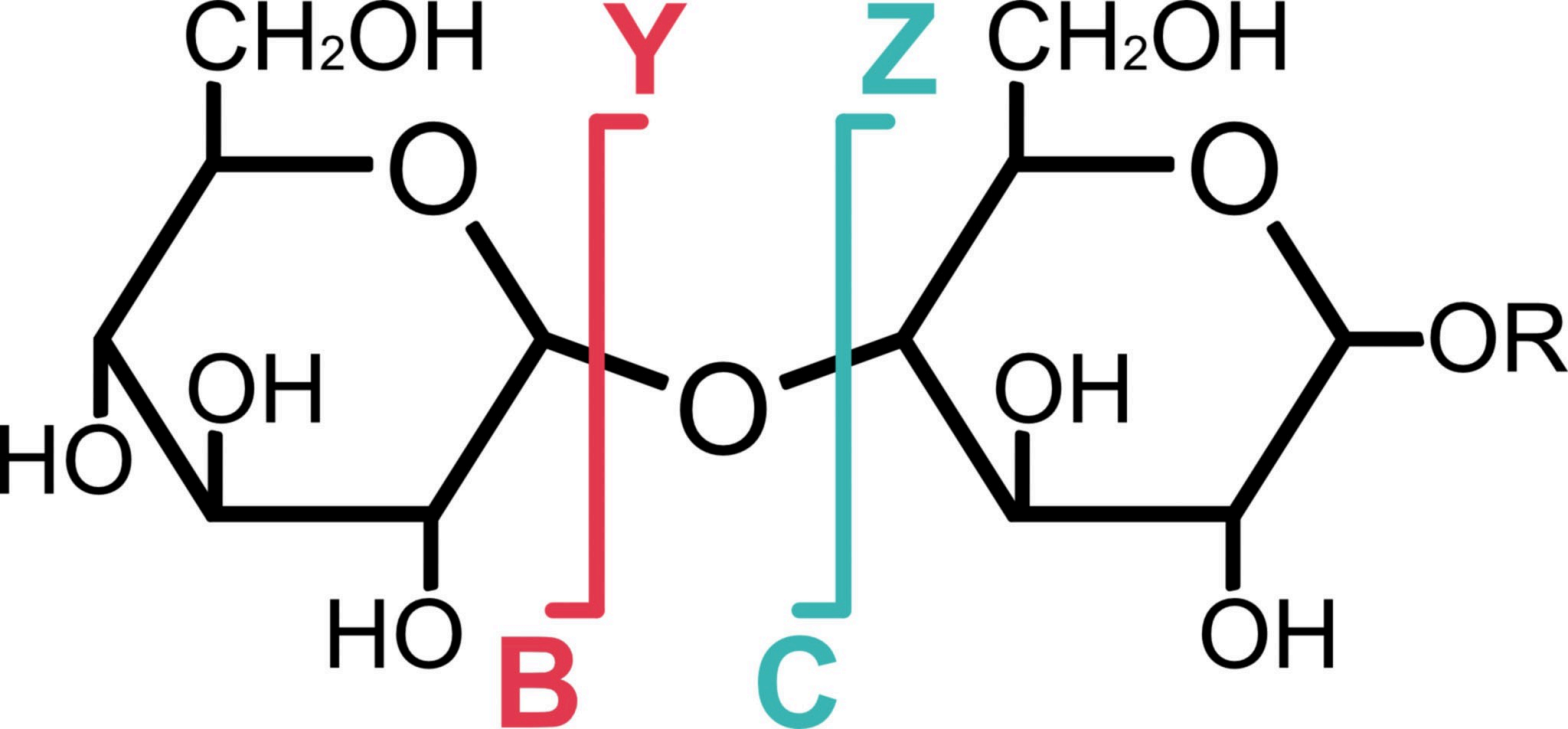
Schematic representation of the Domon and Costello nomenclature^8^ for glycan fragmentation in mass spectrometry. Oligosaccharides can fragment into various smaller ions. B‐type fragments correspond to glycosyl cations, which play an important role in glycosynthesis. Cross‐ring A‐ and X‐fragments have been omitted for clarity.

Glycosidic B‐type fragment ions are in many ways similar to the glycosyl cations in the above‐mentioned mechanistic studies. The released leaving group resembles the corresponding Y‐type fragment at the non‐reducing end. In both synthesis and MS‐based sequencing it is crucial to understand if and in how far the leaving group anomericity influences the structure of the resulting fragment/glycosyl cation. To address this issue, we here analysed a series of B‐fragments from protected monosaccharide precursors that differ in the type and anomericity of the leaving group using cryogenic vibrational spectroscopy and IM‐MS using experimental setups described previously.^9^

Briefly, glycosyl cations are generated by nanoelectrospray ionisation (nESI) and subsequent in‐source fragmentation of monosaccharide precursors bearing thioethyl (SEt) or trichloroacetimidate (TCAI) leaving groups of defined stereochemistry (see the Supporting Information for MS spectra). After *m/z*‐selection, the ions are collected in a hexapole ion trap that allows for buffer gas cooling of the ions to 90 K. Next, superfluid helium nanodroplets traverse the ion trap, picking up analyte ions that are rapidly cooled to 0.4 K in the superfluid helium matrix. The captured ions are guided to a detection region, where the beam of helium droplets overlaps with IR radiation (1000–1800 cm^−1^), produced by the Fritz Haber Institute free‐electron laser (FHI FEL).^10^ The subsequent absorption of resonant IR photons leads to the release of bare intact ions from the nanodroplet. The ion yield is measured with a time‐of‐flight detector and plotted against the wavenumber, leading to highly resolved IR spectra of the investigated ions. Ion mobility measurements to explore the overall shape of the ions were carried out using a home‐built drift‐tube (DT) instrument.^11^ The measured drift times in helium buffer gas were converted into collision cross sections (^DT^CCS_He_)^12^ using the Mason‐Schamp equation.^13^

Initially, the influence of the leaving group on the structure of glycosyl cations was probed. Cryogenic IR signatures and CCSs of B‐type fragments of 4,6‐di‐*O*‐acetyl‐2,3‐di‐*O*‐benzyl‐d‐galactopyranoside precursors with β‐SEt (**1**, Figure 1a) and β‐TCAI (**2**, Figure 1b) leaving groups were recorded. The spectra show six well‐resolved absorption bands. Features below 1300 cm^−1^ are attributed to complex C−O and C−C stretching vibrations and depict a fingerprint, which is unique for structurally distinct analytes.4b The bands above 1300 cm^−1^ are usually diagnostic for functional groups. Here, they result from the symmetric and the antisymmetric dioxolenium stretch ν(COO^+^) as well as the carbonyl stretch ν(C=O).5a

**Figure 1:**
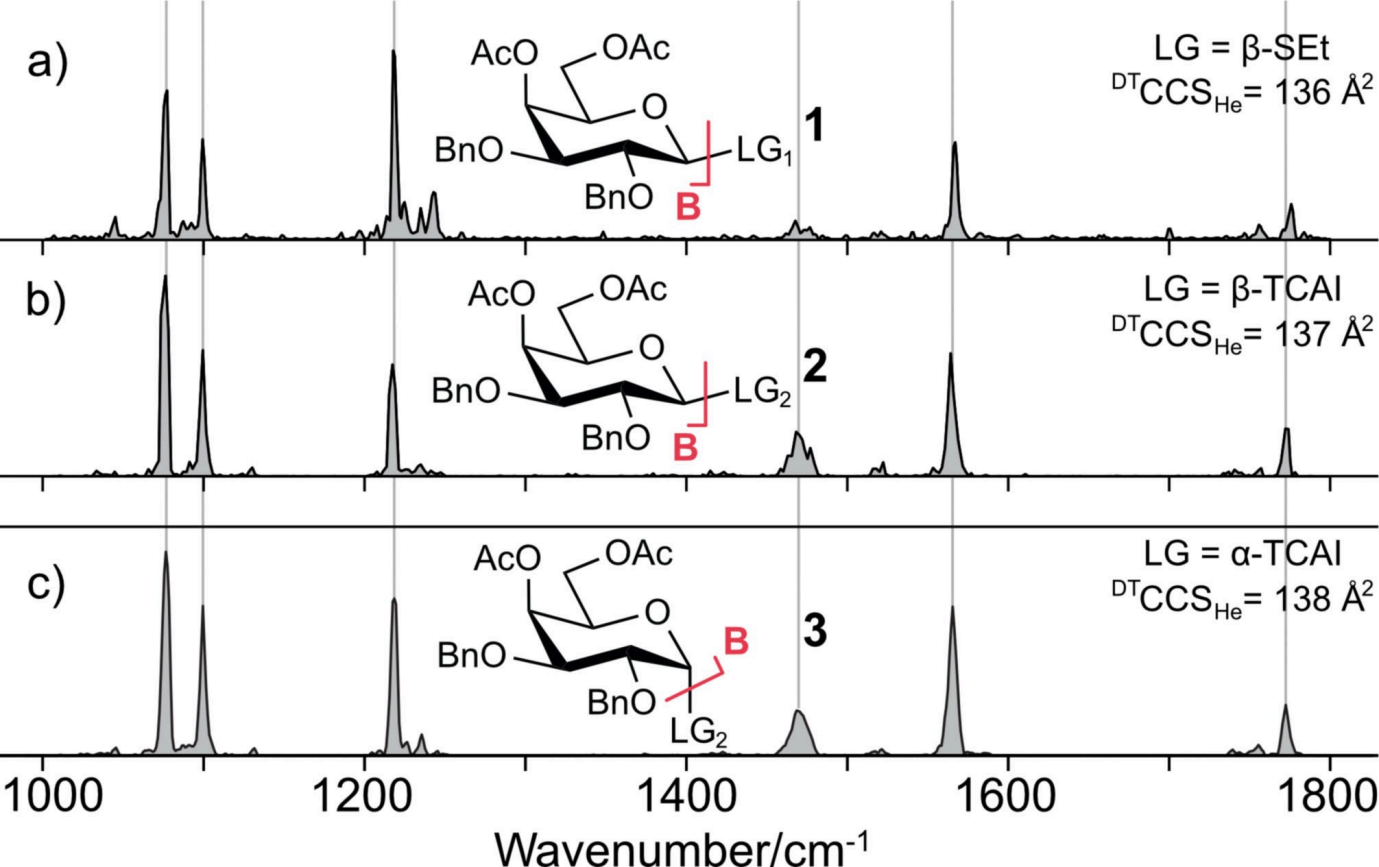
Impact of the leaving group on the structure of B‐type ions. Infrared spectra of glycosyl cations generated from a) β‐thioethyl (**1**, SEt), b) β‐ (**2**) and c) α‐trichloroacetimidate (**3**, TCAI) precursors of 4,6‐di‐*O*‐acetyl‐2,3‐di‐*O*‐benzyl‐d‐galactopyranoside. Regardless of the chemical type and anomericity of the leaving group, similar IR signatures are obtained. Ac: acetyl; Bn: benzyl; LG: leaving group, ^DT^CCS_He_: Drift‐tube collision cross section in He.

Generally, the signatures are identical. However, the spectra of B‐fragments from β‐SEt precursors exhibit a slightly lower signal‐to‐noise ratio than the β‐TCAI counterpart. The similarities of the obtained IR spectra indicate that the ions generated from both precursors exhibit an identical structure. If the B‐fragments were structurally different, then at least variations in the fingerprint region (ca. 1000–1150 cm^−1^) are expected, as even subtle changes in the chemical environment are known to cause a shift of the absorption bands.4b A structural similarity is further supported by the CCSs of the probed glycosyl cations. We conclude that the chemical structure of the leaving group has no influence on the structure of the analysed fragments. Next, the influence of the leaving group anomericity was studied. The CCSs and IR signatures of B‐fragments generated from the intact precursor monosaccharide bearing β‐TCAI (**2**, Figure 1b) and α‐TCAI (**3**, Figure 1c) leaving groups were recorded. The CCSs show minute variations that are within the error of the method, and the vibrational spectra are identical. These results clearly show that the structure of the investigated B‐type ions is not dependent on the leaving group anomericity. As a consequence, these ions do not exhibit an anomeric memory.

To further verify these findings, we applied our methodology to B‐fragments generated from 4‐*O*‐acetyl‐2,3,6‐tri‐*O*‐benzyl‐d‐galactopyranoside (4 and 5) and 6‐*O*‐acetyl‐2,3,4‐tri‐*O*‐benzyl‐d‐galactopyranoside (6 and 7) precursors. In addition to differences in the leaving group and its anomericity, these building blocks also differ in the position of the acetyl protecting group, which is located either at C4 or C6. CCSs and IR spectra of B‐fragments of precursors bearing β‐SEt (4 and 6, Figure 2a and 2c) and α‐TCAI (5 and 7, Figure 2b and 2d) leaving groups were recorded. Within each set, the CCSs are almost identical. Also, the IR signatures of the B‐type fragments exhibit identical absorption bands for each individual set, whereas there are considerable differences between the spectra originating from structurally different glycosyl cations. This result demonstrates that the method is highly diagnostic to structural differences, for example in protecting group regiochemistry. It can therefore be concluded that the structures of B‐type ions of each individual set (i. e. 1, 2, and 3) are virtually identical and independent of the leaving group and its underlying anomericity.

**Figure 2:**
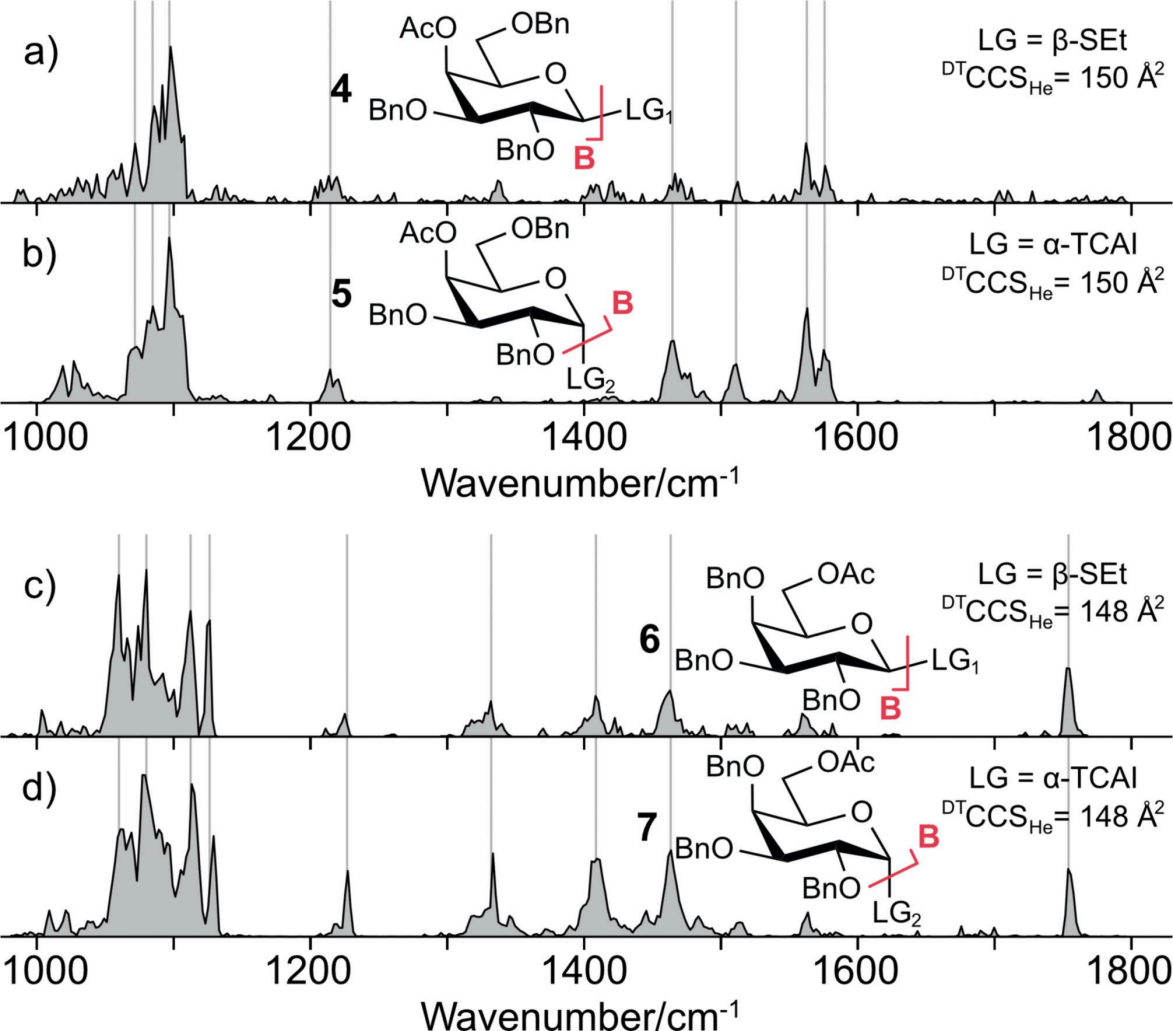
Influence of the leaving group anomericity on the structure of B‐type ions. Infrared spectra of glycosyl cations from a) β‐thioethyl (**4**), b) α‐trichloroacetimidate (**5**) precursors of 4‐*O*‐acetyl‐2,3,6‐tri‐*O*‐benzyl‐d‐galactopyranoside and c) β‐thioethyl (**6**), d) α‐trichloroacetimidate (**7**) precursors of 6‐*O*‐acetyl‐2,3,4‐tri‐*O*‐benzyl‐d‐galactopyranoside. The IR signature of the respective B‐type fragment is not dependent on the leaving group of the precursor. Ac: acetyl; Bn: benzyl; LG: leaving group, ^DT^CCS_He_: Drift‐tube collision cross section in He.

In conclusion, our data show that the chemical nature of the leaving group as well as the stereochemistry of their linkage do not have an impact on the conformation of the resulting glycosyl cation. This implies that regardless of the type and anomericity of the leaving group, reactive intermediates of similar structure are formed. At the same time, our data also indicate that glycosidic B‐type fragments do not exhibit an anomeric memory that was previously shown for C‐type fragments.^6^ It is important to point out that all monosaccharides studied here are fully protected and do not contain free hydroxyl groups. As these groups are crucial for the three‐dimensional structure of glycan fragments, an anomeric memory in unprotected B‐type fragments cannot be ruled out based on the present data. Our data confirm previous studies^7^ on permethylated glycans, which have suggested that hydroxyl groups may play an important role in the anomeric memory effect.

## Conflict of interest

The authors declare no conflict of interest.

## Supporting information

As a service to our authors and readers, this journal provides supporting information supplied by the authors. Such materials are peer reviewed and may be re‐organized for online delivery, but are not copy‐edited or typeset. Technical support issues arising from supporting information (other than missing files) should be addressed to the authors.

Supplementary
