# Supplementary material for: The Impact of Leaving Group Anomericity on the Structure of Glycosyl Cations of Protected Galactosides
Source: Chemphyschem. 2020 Jul 30;21(17):1905–7. doi: 10.1002/cphc.202000473 (PMC7540451; doi:10.1002/cphc.202000473)
Supplement: Supplementary file 1 — Supplementary [file CPHC-21-1905-s001.pdf]

ChemPhysChem

Supporting Information

The Impact of Leaving Group Anomericity on the Structure of Glycosyl Cations of Protected Galactosides

Kim Greis, Eike Mucha, Maike Lettow, Daniel A. Thomas, Carla Kirschbaum, Sooyeon Moon, Alonso Pardo-Vargas, Gert von Helden, Gerard Meijer, Kerry Gilmore, Peter H. Seeberger, and Kevin Pagel*

Experimental Details

Synthesis

The synthesis of the β-thioethyl, β-trichloroacetimidate and α-trichloroacetimidate precursors of 4,6-di-*O*-acetyl-2,3-di-*O*-benzyl-D-galactopyranoside, 4-*O*-acetyl-2,3,6-tri-*O*-benzyl-D-galactopyranoside and 6-*O*-acetyl-2,3,4-tri-*O*-benzyl-D-galactopyranoside has been described previously.[1]

Mass Spectrometry and Infrared (IR) Spectroscopy

The precursors were dissolved in acetonitrile and water (9:1, V:V) to yield 0.1 mM solutions. The samples were ionised *via* nano electrospray ionisation (nESI) with Pd/Pt coated glass capillaries (Sputter Coater HR 208, *Cressington*), which are pulled to a tip with an inner diameter of 1–2 µm using a micropipette puller (Model P-1000, *Sutter Instrument*).

Mass and IR spectra (Figures S1–S7 and 2–3) were recorded using a custom helium droplet instrument, which is briefly described in the following paragraphs. Both capillary and Z-spray sources were used to generate bare glycosyl cations in the gas phase, after employing a voltage of 1 kV to the tip of the capillary (nanoelectrospray ionisation).

A quadrupole mass filter allowed mass-to-charge selection of the glycosyl cations. Subsequently, a hexapole ion trap was filled with the selected ions, which were then thermalised by collisions with buffer gas. The ion trap was cooled with liquid nitrogen to 90 K.

A pulsed Even-Lavie valve was used to generate a beam of superfluid helium droplets by expansion of ^{4}He from high pressure and low temperature into vacuum. The droplets traversed the ion trap, where they picked up ions and rapidly cooled them to 0.4 K. Then the ions were guided to a detection region, where the beam of helium droplets overlapped with an IR beam of the Fritz Haber Institute free-electron laser (FHI FEL[2]), exciting vibrational modes of the analyte ions. The vibrational energy of the embedded ions dissipated through the helium matrix, which subsequently evaporated until bare analyte ions were released and guided to a time-of-flight detector. The ion count as a function of the wavenumber of the IR pulse leads to an IR spectrum.

Mass Spectra

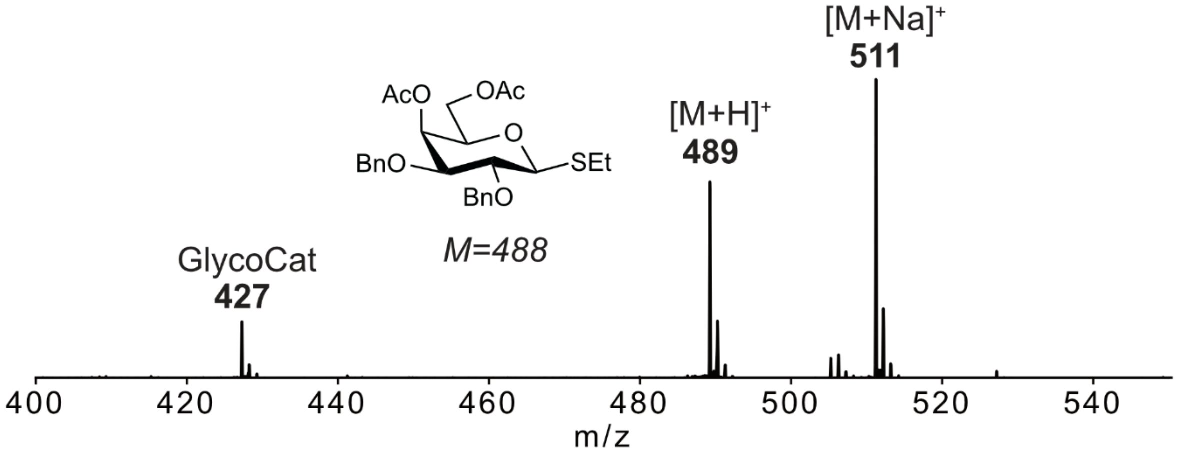

Figure S1: Mass spectrum of 4,6-di-O-acetyl-2,3-di-O-benzyl-D-galactopyranoside generated from β-thioethyl (**1**) precursor recorded on the helium droplet instrument. Glycosyl cations (*m*/*z* = 427) are generated by in-source fragmentation of the precursor ions [M+H]$^+$ (*m*/*z* = 489) and [M+Na]$^+$ (*m*/*z* = 511).

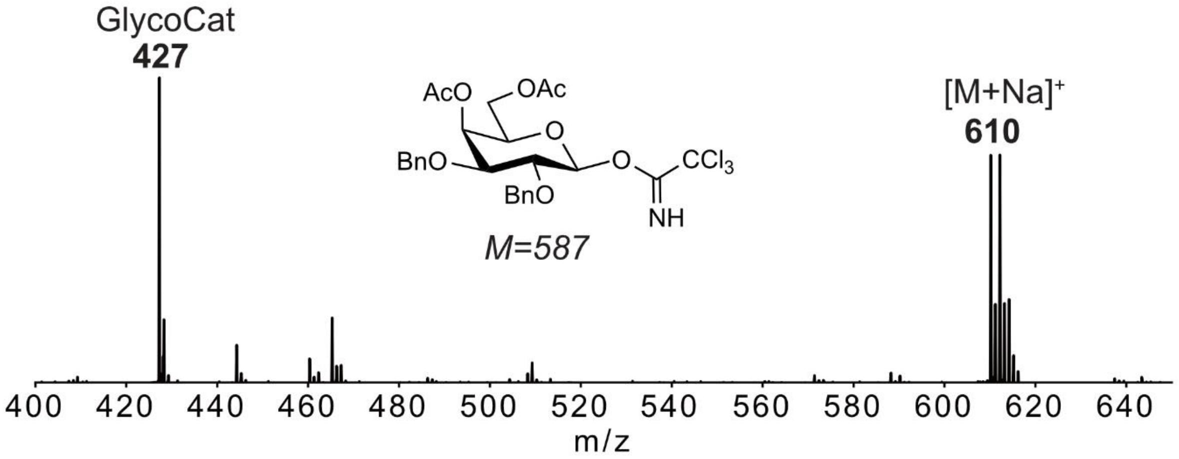

Figure S2: Mass spectrum of 4,6-di-O-acetyl-2,3-di-O-benzyl-D-galactopyranoside generated from β-trichloroacetimidate (**2**) precursor recorded on the helium droplet instrument. Glycosyl cations (*m*/*z* = 427) are generated by in-source fragmentation of the precursor ion [M+Na]$^+$ (*m*/*z* = 610).

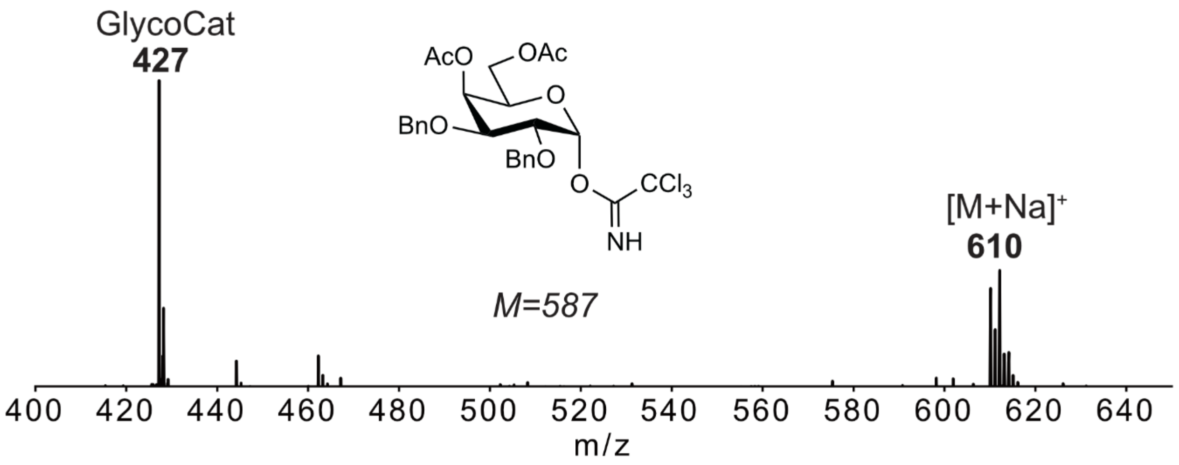

Figure S3: Mass spectrum of 4,6-di-O-acetyl-2,3-di-O-benzyl-D-galactopyranoside generated from α-trichloroacetimidate (**3**) precursor recorded on the helium droplet instrument. Glycosyl cations (*m*/*z* = 427) are generated by in-source fragmentation of the precursor ion [M+Na]$^+$ (*m*/*z* = 610).

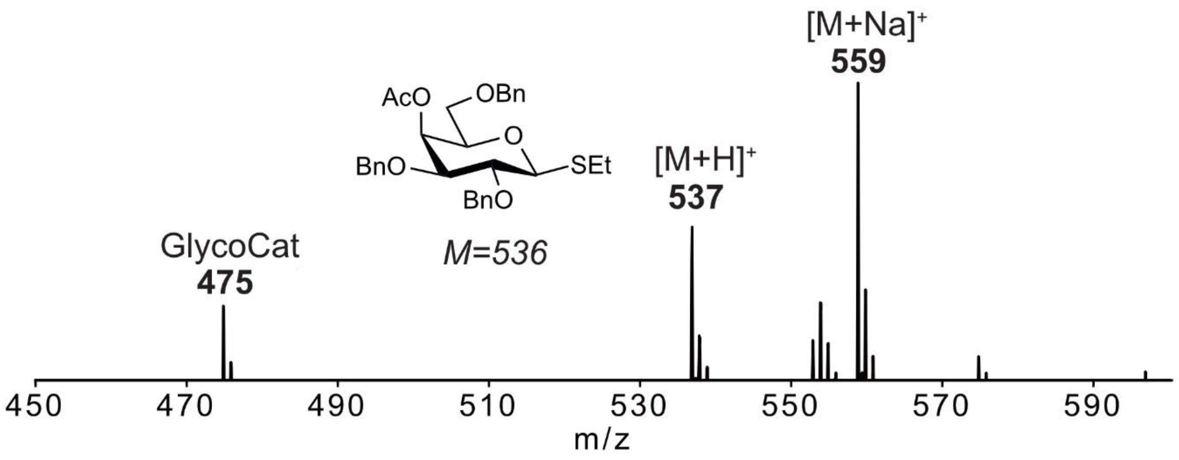

Figure S4: Mass spectrum of 4-O-acetyl-2,3,6-tri-O-benzyl-D-galactopyranoside generated from β-thioethyl (**4**) precursor recorded on the helium droplet instrument. Glycosyl cations (*m*/*z* = 475) are generated by in-source fragmentation of the precursor ions [M+H]$^+$ (*m*/*z* = 537) and [M+Na]$^+$ (*m*/*z* = 559).

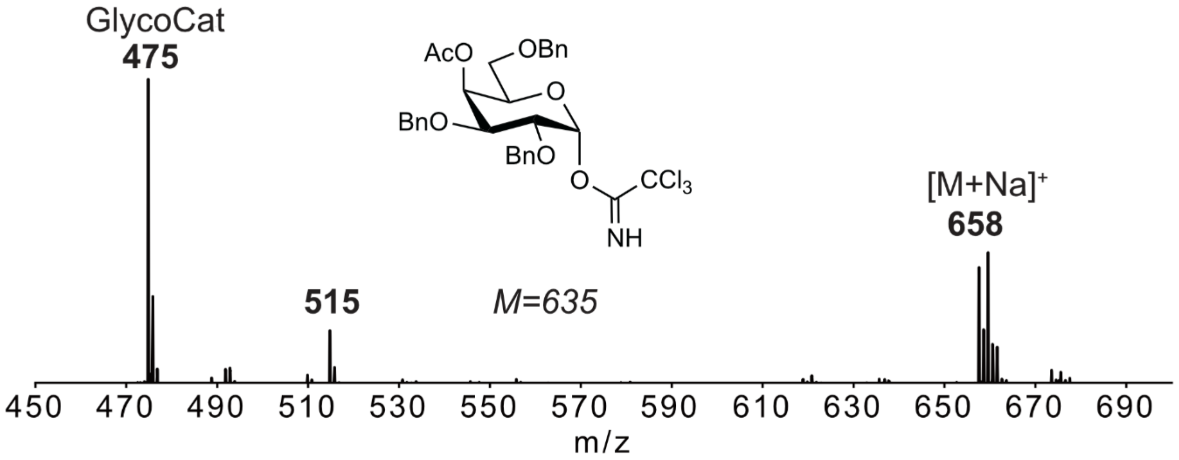

Figure S5: Mass spectrum of 4-O-acetyl-2,3,6-tri-O-benzyl-D-galactopyranoside generated from α-trichloroacetimidate (**5**) precursor recorded on the helium droplet instrument. Glycosyl cations (*m*/*z* = 475) are generated by in-source fragmentation of the precursor ion $[M+Na]^+$ (*m*/*z* = 658).

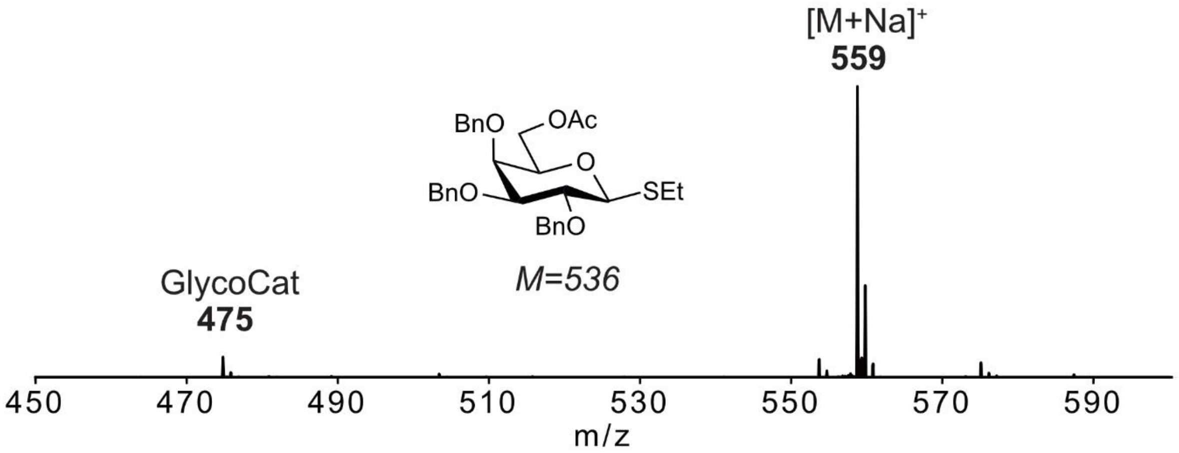

Figure S6: Mass spectrum of 6-O-acetyl-2,3,4-tri-O-benzyl-D-galactopyranoside generated from β-thioethyl (**6**) precursor recorded on the helium droplet instrument. Glycosyl cations (*m*/*z* = 475) are generated by in-source fragmentation of the precursor ion $[M+Na]^+$ (*m*/*z* = 559).

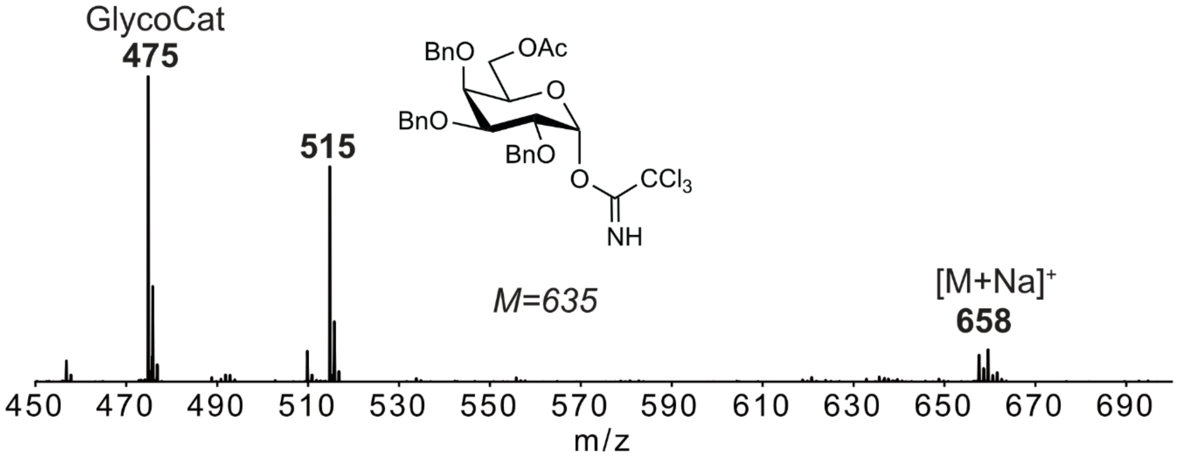

Figure S7: Mass spectrum of 6-O-acetyl-2,3,4-tri-O-benzyl-D-galactopyranoside generated from α-trichloroacetimidate (**7**) precursor recorded on the helium droplet instrument. Glycosyl cations (*m*/*z* = 475) are generated by in-source fragmentation of the precursor ion $[M+Na]^+$ (*m*/*z* = 658).

Ion Mobility–Mass Spectrometry

Solutions and capillaries were prepared as described for recording MS and IR spectra. The drift times were recorded using a home-built drift-tube ion mobility instrument that will be briefly described in the following paragraph.[3]

Glycosyl cations were generated by nanoelectrospray ionisation using a capillary source. The ions were injected to a drift tube (80.55 cm) filled with helium (ca. 4.00 mbar). Drift times were recorded at eleven distinct drift voltages from 1200 to 700 V in 50 V steps. To ensure reproducibility, the measurements were repeated on two different days. Collision cross sections (CCSs) were derived from the measured drift times using a modified Mason-Schamp equation (1).[4, 5] The double standard deviation of each measurement is lower than 1 % of the absolute CCS. The arrival time distributions (ATDs) at 1200 V for each precursor are shown in Figures S8.

The ATDs of the glycosyl cations generated from **6** and **7** suggest the presence of two structures exhibiting a clearly distinct drift time. The wealth of signals in the IR spectrum of these species also suggests that it stems from multiple structures. In a previous study[1] it has been determined that the signals at 1221 and 1561 cm^{-1} are diagnostic for oxocarbenium type structures while it has not been possible to assign the three intense absorption bands between 1300 and 1500 cm^{-1}. In a recent study[6], Hansen *et al.* found out that similar glycosyl cations carrying methyl instead of benzyl protecting groups spontaneously undergo ring opening in the gas phase. The copresence of oxocarbenium and ring-opened structures might explain the composite feature in the ATDs for glycosyl cations generated from **6** and **7**.

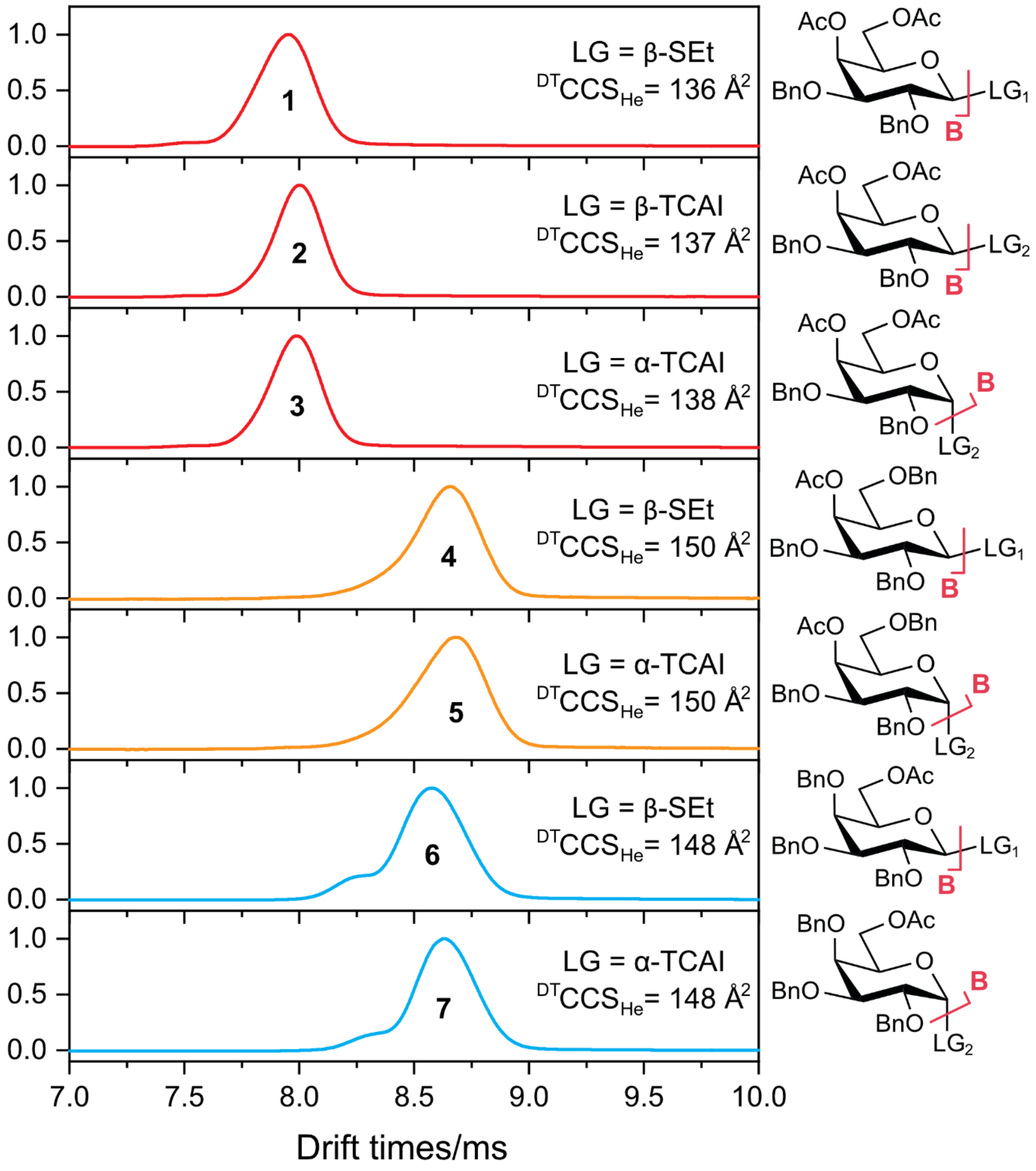

Figure S8: Normalised arrival time distributions (ATDs) of the glycosyl cations 4,6-di-O-acetyl-2,3-di-O-benzyl-D-galactopyranoside (red) generated from β-thioethyl (**1**), β-trichloroacetimidate (**2**) or α-trichloroacetimidate (**3**) precursors, 4-O-acetyl-2,3,6-tri-O-benzyl-D-galactopyranoside (orange) generated from β-thioethyl (**4**) or α-trichloroacetimidate (**5**) precursors and 6-O-acetyl-2,3,4-tri-O-benzyl-D-galactopyranoside (blue) generated from β-thioethyl (**6**) or α-trichloroacetimidate (**7**) precursors. The structures are depicted on the right-hand side of the ATDs. The leaving group and its anomericity do not have a significant impact on the drift times and resulting collision cross sections of the formed glycosyl cations.

References

1. M. Marianski, E. Mucha, K. Greis, S. Moon, A. Pardo, C. Kirschbaum, D. A. Thomas, G. Meijer, G. von Helden, K. Gilmore, P. H. Seeberger and K. Pagel, *Angew. Chem. Int. Ed.*, 2020, **59**, 6166-6171.
2. W. Schöllkopf, S. Gewinner, H. Junkes, A. Paarmann, G. von Helden, H. P. Bluem and A. M. M. Todd, *Proc. SPIE Int. Soc. Opt. Eng.*, 2015, 95121L.
3. S. Warnke, G. von Helden and K. Pagel, *Proteomics*, 2015, **15**, 2804-2812.
4. E. A. Mason and E. W. McDaniel, *Transport Properties of Ions in Gases*, John Wiley & Sons, New York, United States, 1988.
5. H. E. Revercomb and E. A. Mason, *Anal. Chem.*, 2002, **47**, 970-983.
6. T. Hansen, H. Elferink, J. M. A. van Hengst, K. J. Houthuijs, W. A. Remmerswaal, A. Kromm, G. Berden, S. van der Vorm, A. M. Rijs, H. S. Overkleeft, D. V. Filippov, F. Rutjes, G. A. van der Marel, J. Martens, J. Oomens, J. D. C. Codee and T. J. Boltje, *Nat. Commun.*, 2020, **11**, 2664.
